# Cobra Venom Factor and Ketoprofen Abolish the Antitumor Effect of Nerve Growth Factor from Cobra Venom

**DOI:** 10.3390/toxins9090274

**Published:** 2017-09-06

**Authors:** Alexey V. Osipov, Tatiana I. Terpinskaya, Tatiana E. Kuznetsova, Elena L. Ryzhkovskaya, Vladimir S. Lukashevich, Julia A. Rudnichenko, Vladimir S. Ulashchyk, Vladislav G. Starkov, Yuri N. Utkin

**Affiliations:** 1 Shemyakin-Ovchinnikov Institute of Bioorganic Chemistry, Russian Academy of Sciences, ul. Miklukho-Maklaya 16/10, Moscow 117997, Russia; osipov@mx.ibch.ru (A.V.O.); vladislavstarkov@mail.ru (V.G.S.); 2 Institute of Physiology, National Academy of Sciences of Belarus, ul. Akademicheskaya, 28, Minsk 220072, Belarus; terpinskayat@mail.ru (T.I.T.); tania_k@mail.ru (T.E.K); ryzhkovskaya@mail.ru (E.L.R.); lukashvs@rambler.ru (V.S.L); link060619@list.ru (J.A.R); ulashchikv@mail.ru (V.S.U.)

**Keywords:** Ehrlich carcinoma, immune system, nerve growth factor, cobra venom, cobra venom factor, ketoprofen

## Abstract

We showed recently that nerve growth factor (NGF) from cobra venom inhibited the growth of Ehrlich ascites carcinoma (EAC) inoculated subcutaneously in mice. Here, we studied the influence of anti-complementary cobra venom factor (CVF) and the non-steroidal anti-inflammatory drug ketoprofen on the antitumor NGF effect, as well as on NGF-induced changes in EAC histological patterns, the activity of lactate and succinate dehydrogenases in tumor cells and the serum level of some cytokines. NGF, CVF and ketoprofen reduced the tumor volume by approximately 72%, 68% and 30%, respectively. The antitumor effect of NGF was accompanied by an increase in the lymphocytic infiltration of the tumor tissue, the level of interleukin 1β and tumor necrosis factor α in the serum, as well as the activity of lactate and succinate dehydrogenases in tumor cells. Simultaneous administration of NGF with either CVF or ketoprofen abolished the antitumor effect and reduced all other effects of NGF, whereas NGF itself significantly decreased the antitumor action of both CVF and ketoprofen. Thus, the antitumor effect of NGF critically depended on the status of the immune system and was abolished by the disturbance of the complement system; the disturbance of the inflammatory response canceled the antitumor effect as well.

## 1. Introduction

Nerve growth factor (NGF) belongs to a protein family of neurotrophins, which are important agents affecting the cell cycle. Usually they are mitogens; mammalian NGF has evident regenerative (primarily neuroprotective) functions. Among prevailing highly toxic ingredients, snake venoms contain non-toxic NGF. NGF from snake venom is a polypeptide possessing homology with more than 80% of identical amino acid residues to the beta-chain of NGF from mammalian saliva [1]; This phenomenon is explained by the origin of the snake venom glands from the salivary glands.

NGF can bind to two classes of receptors on the cell surface: the TrkA receptor, a tyrosine kinase with high specificity and high affinity to NGF, and p75, a low-affinity neurotrophin receptor which can bind to all members of the neurotrophin family with approximately equal affinity. NGF binding to the TrkA receptor triggers a number of metabolic reactions through a cascade of protein kinases. There are some data about the involvement of NGF in carcinogenesis [2], and mammalian NGF may promote or suppress tumor growth, depending on the tumor type. Snake venom NGF exerts some activities characteristic of mammalian NGF; however, the data concerning pro- or anti-oncogenic activity of snake NGF are very limited. We have recently shown that NGF from cobra venom may exert an inhibitory effect on the growth of Ehrlich’s adenocarcinoma in vivo [3]. This effect is mediated via a tyrosine kinase cascade, because it can be blocked by the tyrosine kinase inhibitor K252a [4].

Considering the tumor as a disease at the level of the whole organism, we should take into consideration the role of the tumor microenvironment and the overall condition of the homeostasis systems, primarily the immune system. Tumor growth in the body is accompanied by an inflammatory reaction that, on the one hand, is aimed to eliminate tumor cells, and on the other hand, induces a tolerance to the tumor maintenance and growth [5].

One of the systems involved in the development of tumor-associated inflammation is complement. The accumulation of the complement C5 component with the subsequent generation of C5A anaphylatoxin by complement C3/C5 convertase contributes to the immunosuppressive properties of the tumor microenvironment [6]. Cobra venoms contain an activator of alternative pathway of the complement system, the so-called cobra venom factor (CVF), which is similar to the complement C3b component—forming C3/C5 convertase. However, CVF is not regulated by complement components, and activates the system in such a way that depletes it rapidly and completely. We have shown earlier that CVF from the Thailand cobra venom affects subcutaneous Ehrlich carcinoma in mice [7].

The activity of cyclooxygenases, in particular, cyclooxygenase-2 (COX-2), has proved to be essential for the development of chronic inflammation, tumor growth and metastasis during carcinogenesis [8,9]. Use of nonsteroidal anti-inflammatory drugs (NSAIDs) leads to a decrease of tumor size [8,9]. The NSAID ketoprofen has been shown to inhibit tumor cell proliferation in vitro [10] and in vivo [11,12].

Thus, CVF and ketoprofen, through various mechanisms, counteract the development of an inflammatory tumor microenvironment. To check if these drugs act synergistically with NGF, we have studied the influence of CVF and ketoprofen on the antitumor effects of cobra venom NGF in vivo. To see the effects at the tissue level, we have undertaken histological investigations of tumor samples. The concentration of tumor growth factor β1 (TGF-β1), interleukin 1β (IL-1β) and tumor necrosis factor α (TNF-α) in serum of the experimental animals was also determined.

An anomalous characteristic of energy metabolism in cancer cells is accelerated glycolysis, even in the presence of oxygen, rather than mitochondrial oxidative phosphorylation (the Warburg effect). It is accompanied by an elevated activity of lactate dehydrogenase (LDH, EC 1.1.1.27), which catalyzes the conversion of pyruvate to lactate. However, some sub-populations of cancer cells display high mitochondrial respiration and low glycolysis rates [13]. One of the key enzymes in aerobic oxidation is succinate dehydrogenase (SDH, EC 1.3.99.1). To determine the energy processes ongoing in EАC, the influence of NGF, CVF and ketoprofen on the activity of LDH and SDH have been studied.

Here, we report that simultaneous administration of either CVF or ketoprofen with NGF cancels antitumor effects and reduces other effects of NGF in Ehrlich ascites carcinoma (EAC)-bearing mice.

## 2. Results

To study effects of NGF, CVF, ketoprofen and combinations thereof, the mice were grafted subcutaneously with EAC, as described earlier [4]. Three experimental inoculated groups were injected intraperitoneally (i.p.) with either NGF, CVF or ketoprofen. Two other experimental inoculated groups were injected with a combination of NGF with either CVF or ketoprofen.

### 2.1. The Effects on Ehrlich Carcinoma Growth

**Figure 1:**
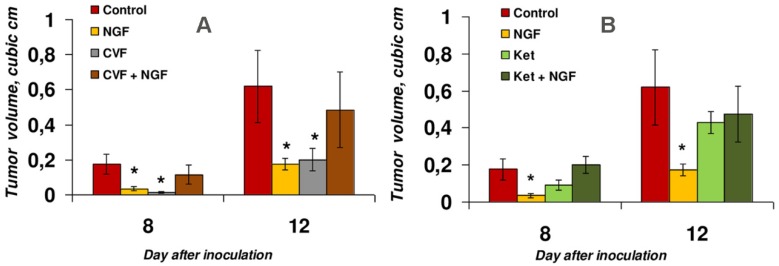
The effects of NGF and its combination with either CVF (**A**) or ketoprofen (Ket) (**B**) on Ehrlich ascites carcinoma (EAC) growth. * *p* < 0.05 compared to control (Mann–Whitney test).

The effects were analyzed at days eight and twelve after inoculation (Figure 1). NGF inhibited the tumor growth by about 72% (columns two in Figure 1A,B), which was in accordance with our previously published data [4].

When administered alone, CVF had an in vivo antitumor effect comparable to that of NGF (Figure 1A, column three) which resulted at day 12 in a reduction of mean tumor size by 68% compared to the control. However, combined administration of NGF and CVF critically reduced the antitumor effect of both drugs. Although a decrease in the tumor volume by 22% was observed at day twelve after inoculation (and by 35% at day eight), it was statistically insignificant according to the Mann–Whitney test (Figure 1A, column four). This points to the importance of the normal functioning of the immune system, in particular, the humoral system, for the antitumor effect of NGF. These data correlate well with our preliminary results [7].

Ketoprofen showed a tendency to decrease the tumor size by 48% (statistically insignificant according to the Mann–Whitney test) at day eight (Figure 1B, column three) after inoculation. This tendency was in agreement with the data of da Silveira et al. [12] obtained on glioma-bearing rats, and to the data of Sakayama et al. [11] on pulmonary metastasis of LM8 cells observed in male nude mice. In our work, at day twelve, the average tumor size reduction was only 30%, with a wide variation of this parameter in individual animals. Ketoprofen abolished the antitumor effect of NGF on both day eight and day twelve (Figure 1B, column four). Given that COX-1 and COX-2 are the main targets for ketoprofen [14], our data suggested that, at the level of regulation by cyclooxygenases, the normal manifestation of the inflammatory response is necessary for the antitumor effect of NGF.

### 2.2. Histological Studies of Tumor Samples

To determine the effects produced by NGF, CVF and ketoprofen, as well as NGF in combination with CVF or ketoprofen, in EAC at the tissue level, histological studies of tumor samples were carried out. Slices of tumor tissues, obtained at day twelve, were stained with hematoxylin–eosin and analyzed by microscopy.

**Figure 2:**
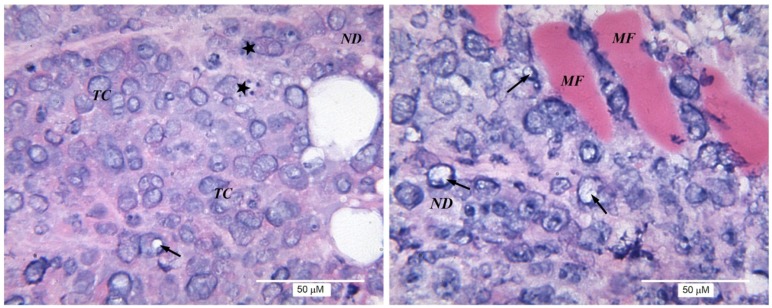
Morphological structure of EAC. For representation of different structures, two panels displaying separate parts of the same tumor are shown. Here, and in Figure 3 and Figure 4, stars indicate lymphocytes; arrows: karyorrhexis or vacuolization of nuclei; MF: muscle fibers (in control: smooth, when exposed to NGF: striated); TC: tumor cells; and ND: necrotic detritus.

Evident cellular polymorphism was observed in the tumor tissue. Cells with destructive changes, with vacuolization of the nuclei and cytoplasm, and giant multinucleated cells, were found in most of the tumor nodules. In tumor sections from the untreated control series (Figure 2), both minor structural changes and the destruction of a significant number of tumor cells were observed. Immune cell infiltration was absent or was poorly expressed (single scattered lymphocytes and small foci of lymphocytic infiltration).

**Figure 3:**
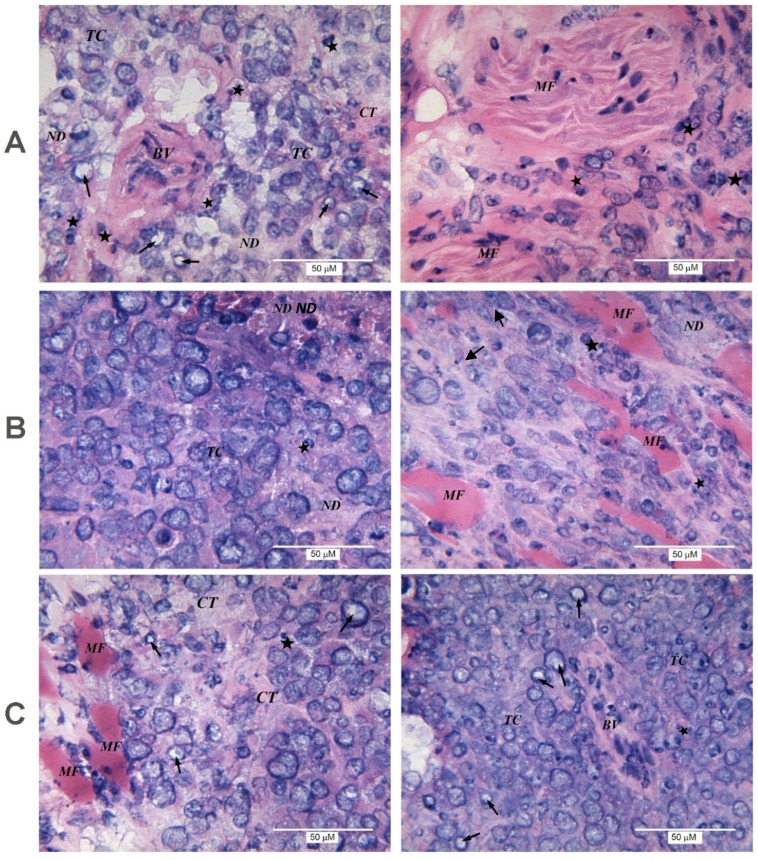
Morphological structure of EAC from mice treated with NGF (**A**) or CVF (**B**) only and with combination of both factors (**C**). CT: connective tissue; BV: blood vessel. Left and right panels show different parts of the same tumor.

When the inoculated mice were treated with NGF, reduction of tumor volume was accompanied by an increase in the number of giant multinucleated cells and areas of unstructured necrotic detritus, as well as by germination of muscle fibers and the presence of large blood vessels in tumor tissue (Figure 3A). The latter indicates that the antitumor effect of NGF cannot be explained by an anti-angiogenic activity. NGF increased the lymphocytic infiltration in the tumor compared to control samples (Figure 3A).

In tumors from mice treated with CVF, the histological analysis revealed no significant differences from the controls. Weak immune cell infiltration was seen (Figure 3B).

The combined administration of NGF and CVF resulted in proliferation of connective tissue. Large blood vessels among nodes of tumor cells (Figure 3C) were typical for the experimental group in which animals received NGF only. Histological analysis of the tumor revealed no significant differences in the pathomorphological features and lymphocytic infiltration from the group that received CVF only, or the control group.

**Figure 4:**
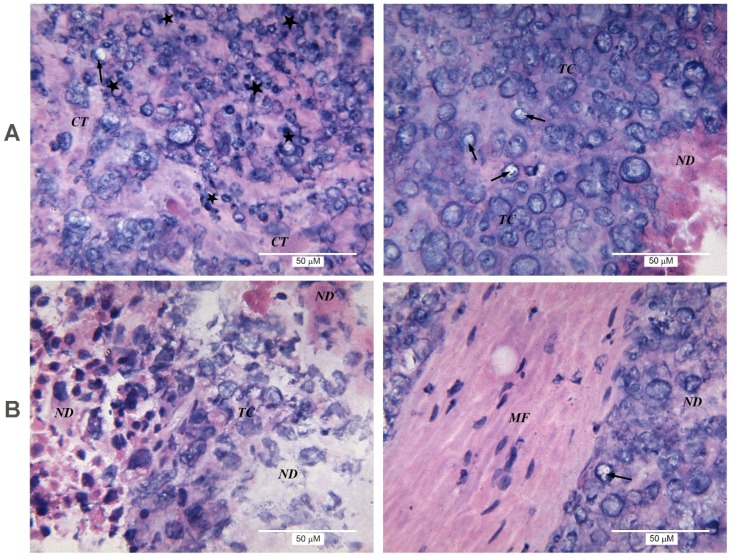
Morphological structure of EAC from mice treated with ketoprofen only (**A**) or with combination of NGF and ketoprofen (**B**). Left and right panels show different parts of the same tumor.

A distinctive feature of the group in which the animals received ketoprofen was a slightly more-pronounced lymphocytic infiltration than in the control—from mild to moderate, with the appearance of medium foci of inflammatory infiltration in the presence of single neutrophils (Figure 4A).

Combined administration of ketoprofen and NGF to inoculated mice resulted in the outgrowth of connective tissue between carcinoma cells. Significant areas of necrotic detritus were seen in the majority of samples (Figure 4B). Similarly to the group treated with NGF only, germination of muscle fibers was detected. Immune cell infiltration was variable, ranging from non-detectable in some tumor slices to weak or moderate in others. In general, lymphocytic infiltration was slightly more pronounced than in the control, but weaker than in the series where mice received ketoprofen or NGF only.

Thus, the increased degree of lymphocytic infiltration was the histological feature of tumors from mice treated with NGF. The application of either CVF or ketoprofen simultaneously with NGF reduced this effect. Moreover, the antitumor activity of CVF was not associated with lymphocytic infiltration, while that of ketoprofen was. Application of NGF alone or in combination with other drugs contributed to the vascularization of tumor nodes, the germination of muscle fibers and the proliferation of connective tissue. In the series where the animals received NGF or NGF with ketoprofen, there was a proliferation of not only smooth muscle, but also striated muscle fibers.

### 2.3. The Effects on TNF-α, IL-1β and TGF-β1 in Serum

**Figure 5:**
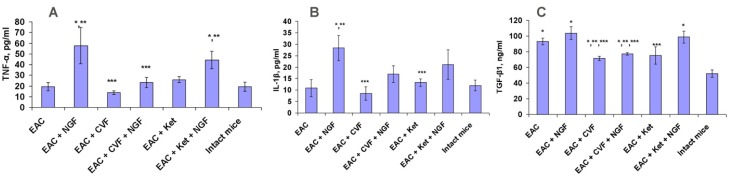
Effect of NGF, CVF or ketoprofen (Ket) on serum levels of TNF-α (**A**), IL-1β (**B**) and TGF-β1 (**C**) in mice bearing EAC. * *p* < 0.05 (compared to intact mice); ** *p* < 0.05 (compared to EAC-bearing mice); *** *p* < 0.05 (compared to EAC-bearing mice treated with NGF).

NGF caused a considerable increase in the level of TNF-α and IL-1β in the serum of the tumor-bearing mice. CVF significantly (and ketoprofen insignificantly) reduced the NGF-induced increase in TNF-α, and similar trends were observed for the level of IL-1β (Figure 5A,B). Therefore, it could be assumed that the antitumor effect of NGF was, to a certain degree, related to the increase of IL-1β and THF-α. Mice with EAC had an increased level of serum TGF-β1; CVF or ketoprofen significantly reduced this level (Figure 5C). NGF preserved the increased TGF-β1 level and canceled the decreasing action of ketoprofen, but not that of CVF (Figure 5C).

### 2.4. Effects on Glycolysis

A characteristic feature of tumor cells is increased glycolysis, which is accompanied by a high activity of LDH; this takes place even under normal oxygenation conditions (aerobic glycolysis). SDH is an enzyme that takes part in the process of aerobic oxidation. Considering these facts, we measured the activity of LDH and SDH as enzymes involved in the carbohydrate and energy metabolism.

**Figure 6:**
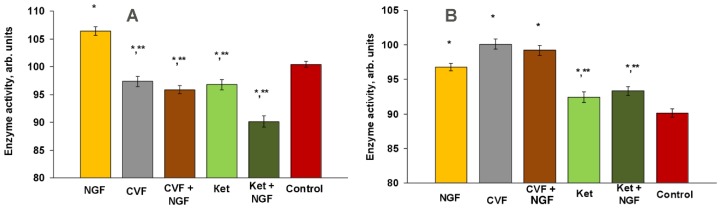
Effect of NGF, CVF and ketoprofen (Ket) on LDH (**A**) and SDH (**B**) activities in EAC (at day 12 after tumor inoculation). * *p* < 0.05 (compared to control), ** *p* < 0.05 (compared to NGF). Control: EAC-bearing mice.

Treatment of tumor-bearing mice with NGF led to an increase in the activity of both LDH and SDH (5.9% and 7.4%, respectively, *p* < 0.05) (Figure 6), i.e., both anaerobic and aerobic oxidation were increased under NGF treatment.

CVF treatment resulted in a decrease of LDH activity (*p* < 0.05) and an increase of SDH activity by 11.1%, *p* < 0.05 (Figure 6). Applied simultaneously, NGF and CVF reduced LDH activity compared to the control (4.5%, *p* < 0.05) and to the group treated with NGF only (9.9%) (column 3 in Figure 6A), and increased SDH activity by 10.1% (*p* < 0.05) compared to the control and by 2.5% (*p* < 0.05) and to the group treated with NGF only (column three in Figure 6B).

Ketoprofen alone reduced LDH activity (3.6%) and increased the activity of SDH (2.4%), shifting the metabolism towards aerobic oxidation (column four in Figure 6A,B). When combined with NGF, ketoprofen neutralized the stimulatory effect of NGF on both SDH and LDH activities (column five versus column one in Figure 6A,B). The reduction in LDH activity was greater by 10.3% than that induced by ketoprofen only (column five versus column four in Figure 6A). The SDH activity after simultaneous application of NGF and ketoprofen was not significantly different from that observed in the presence of ketoprofen only (column five versus column four in Figure 6B).

## 3. Discussion

Long-term intensive studies have shown that the relationship between immunity and cancer is complex [15]. The immune system can excrete factors promoting survival, growth and invasion of tumor cells. Thus, on the one hand, the immune system can act as an extrinsic tumor suppressor, but on the other hand, it facilitates cancer initiation, promotion and progression [15]. The complement system is generally recognized as a protective mechanism against the formation of tumors, but recent studies also indicated a pro-tumorigenic potential of the complement system in certain cancers and under certain conditions [16]. Nevertheless, treatment of tumor-bearing mice with CVF results in a significant growth retardation of B16 melanoma tumors [17] and EAC [7]. It is also known that different NSAIDs, including inhibitors of COX-1 and COX-2 such as ketoprofen, can restrain the development of tumors [8,9]. We have found that ketoprofen suppresses the growth of EAC as well (Figure 1). Earlier, we showed that NGF from cobra venom exerts the same effect on the subcutaneous form of EAC [4]. Based on these data, we decided to check if CVF and ketoprofen would exhibit a synergistic effect with NGF. However, the results obtained showed that neither CVF nor ketoprofen enhanced the antitumor effect of NGF (Figure 1). Instead, both compounds abolished the effect of NGF on EAC (Figure 1). This data indicates that the normal function of the immune system is a prerequisite for the antitumor effect of NGF.

### 3.1. The Inflammatory Infiltration and EAC Growth 

Inflammatory reactions play a key role at different stages of tumor development [18,19,20]. At present, most data underscore the benefits of inflammatory tissue infiltration for tumor progression. One of the potential mechanisms is that chronic inflammation can generate an immunosuppressive microenvironment that benefits tumor formation and progression [21]. However, the balance between antitumor and tumor-promoting immunity can be shifted either to protect against the neoplasia development, or to support tumor growth.

In our experiments, the immune cell infiltrate is mainly composed of lymphocytes. In clinical studies, the increase in lymphocytic infiltration in many (although not all) cases is considered a favorable prognostic sign [22,23]. Our data show that retardation of the EAC growth under NGF treatment is accompanied with an increase in the lymphocyte infiltration (Figure 3). CVF or ketoprofen abolish the NGF effect on EAC, and reduce NGF-induced local lymphocytic infiltration (Figure 3 and Figure 4). Based on these data, one may suggest that the antitumor effect of NGF is mediated by immune cells. At the same time, NGF promotes the sprouting of blood vessels, i.e., it does not display anti-angiogenic properties when exerting its antitumor effect. The promotion of breast cancer angiogenesis by NGF was observed earlier [24]. Such a property is generally considered pro-oncogenic, and in this case might facilitate access of immune cells to the tumor.

In our work, CVF alone has practically no effect on the immune cell infiltration (Figure 3). This finding is in some contradiction with the data of [17], wherein a significant slowdown of the B16 melanoma growth under CVF treatment was accompanied by a significant increase in the number of tumor-infiltrating immune cells. This might be explained by the different schemes of CVF administration used by [17] and in our experiments.

Our data show that lymphocyte infiltration plays some role in tumor development. This issue deserves further study, and may provide new information on the mechanisms of the antitumor effects of NGF, CVF and NSAIDs.

### 3.2. The Levels of TNF-α, IL-1β and TGF-β1 in the Serum of Tumor-Bearing Mice 

An increase in the levels of proinflammatory cytokines IL-1β and TNF-α indicates that the antitumor effect of NGF is realized under conditions of increased inflammation. Ketoprofen, and especially CVF, attenuate or cancel this effect (Figure 5). A decrease in the level of IL-1β and TNF-α by CVF points to the latter’s pronounced anti-inflammatory effect. In turn, the antitumor activity of CVF and ketoprofen, but not NGF, is possibly associated with a decrease in the level of serum TGF-β1, which directs the cells of the tumor microenvironment to the pro-tumor phenotype, and negatively regulates the cytotoxic function of immune cells [25,26].

The antitumor effect of NSAIDs is associated with inhibition of cyclooxygenase activity and a decrease in the synthesis of PGE2, which has a tumor-promoting effect [9,27], and is also associated with a series of cyclooxygenase-independent events [28].

It may be possible that suppression of inflammation prevents the antitumor effect of NGF. Conversely, inflammation induced by NGF may cancel the antitumor effect of CVF.

### 3.3. Glycolysis and EAC Growth

Oxidative stress is considered one of the inducers and markers of carcinogenesis. It contributes to the shift of energy metabolism to enhanced aerobic glycolysis, which is associated with malignant cell proliferation [29], as well as to the enhancement of pro-carcinogenic properties of the tumor cell microenvironment [30]. On the other hand, there is evidence for the "reverse Warburg effect", consisting of a metabolic interaction between glycolytic stroma cells and cancer cells with enhanced oxidative metabolism. Thus, aerobic glycolysis can be characteristic of stromal cells surrounding the tumor, while the tumor cells convert lactate, produced by the stromal cells, into pyruvate, and use the latter in oxidative phosphorylation [13]. 

To study the influence of our compounds on energy metabolism in EAC, we determined the activity of LDH and SDH in the tumor cells (Figure 6). Our data show that the retardation of EAC growth by NGF is accompanied by an increase in both anaerobic and aerobic oxidation in EAC cells. Ketoprofen and CVF influenced NGF-stimulated aerobic oxidation to different extents; ketoprofen significantly reduced the NGF-induced stimulation of aerobic oxidation, while CVF did not. Suppression of the NGF antitumor effect by both CVF and ketoprofen coincided with the suppression by these drugs of the NGF-induced enhancement of glycolysis represented by LDH activity.

Thus, the inhibition of tumor growth after the NGF treatment was accompanied by increased activity of both LDH and SDH. CVF and ketoprofen had different effects on energy metabolism in EAC. Ketoprofen, but not CVF, reduced the increase of aerobic oxidation induced by NGF. However, both significantly reduced the stimulating effect of NGF on anaerobic oxidation (glycolysis).

It should be noted that the activation and cytotoxic response of lymphocytes are associated with an increase in the intensity of glycolysis [31]. One of the possible mechanisms for the antitumor effect of NGF may represent its action on energy metabolism in immune cells. CVF and ketoprofen might also act on energy metabolism in immune cells, however, in a way opposite to that of NGF. Direct experimental confirmation of this suggestion is outside of the scope of this work, and further studies should be performed to address this matter.

### 3.4. The Possible Role of TrkA Receptors

It is known that NGF participates in some inflammatory processes [32]. Thus, various myeloid cells are capable of expressing the NGF receptor TrkA and responding to NGF. For example, about 20% of fresh mouse natural killer (NK) cells express the TrkA receptor, and this fraction reaches 100% when NK cells are activated by interleukin 2. NGF does not influence the expression of surface molecules important for NK cell activation or inhibition. In contrast to TrkA, the other NGF receptor, p75, is not expressed by NK cells (neither resting nor activated) [33]. NGF is involved in eosinophil or B cell survival; its effect may be completely abolished in the presence of K252a, the TrkA receptor antagonist [34,35]. ΝGF can also act as a chemotactic factor for lymphocytes, considering that it mediates attraction of monocytes—other leukocytes from the agranulocyte group—without modifying the production of proinflammatory cytokines [36].

We have earlier shown that K252a can block the NGF inhibitory effect on the growth of EAC [4]. In the present paper, we demonstrate that treatment of mice by NGF leads to increased lymphocyte infiltration of the tumor. Considering these facts, one could suggest that the antitumor effect of NGF is mediated through the TrkA receptor, activation of which on lymphocytes might be more important than on the tumor cells.

## 4. Conclusions

The treatment of tumor-bearing mice with NGF resulted in a reduction of tumor volume by 72%, increased lymphocytic infiltration of the tumor tissue, elevated levels of serum IL-1β and TNF-α and an increase in the activities of both SDH and LDH in EAC cells. Under the same conditions, CVF inhibited the tumor growth by 68%; however, the degree of inflammatory infiltration did not differ significantly from controls. A decrease in the TGF-β1 level in the serum of CVF-treated mice, and a tendency to decrease levels of IL-1β and TNF-α, were observed. With ketoprofen administration, the lymphocytic infiltration was slightly more pronounced; however, the inhibition of tumor growth was less apparent than that induced by CVF. CVF and ketoprofen increased the activity of SDH and decreased LDH activity in tumor cells.

Unlike NGF administration only, the application of NGF together with either ketoprofen or CVF resulted in abrogation of its inhibitory effect on EAC. CVF and ketoprofen decreased lymphocytic infiltration and levels of IL-1β and TNF-α, stimulated by NGF. They also prevented the NGF-induced increase in LDH activity.

Thus, the data obtained in this work suggest that the antitumor effect of NGF in vivo depends critically on the normal status of the immune system. The substances that disturb immunity neutralize the inhibitory effect of NGF on the development of EAC in mice. The NGF antitumor mechanism may cause an increase of lymphocytic infiltration in the tumor, a rise in the levels of IL-1β and TNF-α in the serum of tumor-bearing mice, and an increase in aerobic glycolysis.

## 5. Materials and Methods 

### 5.1. Materials

NGF and CVF were isolated from *Naja kaouthia* cobra venom as described in [37,38], respectively. The purity of samples was more than 98%, as confirmed by analytical reversed-phase HPLC and MALDI mass spectrometry. Ketoprofen in the form of 4% solution for injection was obtained from Pharmaceuticals Lek (Ljubljana, Slovenia).

### 5.2. Mice

Female Af/WySnMv mice were inbreeded at the Institute of Physiology, National Academy of Sciences of Belarus (Minsk, Belarus). EAC was purchased from Blokhin Russian Cancer Research Center, Russian Academy of Medical Sciences (Moscow, Russia). Tumor cells were obtained from the EAC maintained by intraperitoneal passages. All the appropriate actions were taken to minimize discomfort to mice. The World Health Organization’s International Guiding Principles for Biomedical Research Involving Animals were followed during experiments on animals.

### 5.3. Carcinoma Growth and Application of Drugs

Viable EAC cells (20 × 10^6^) were inoculated into the left flank of the mice.

The mice with EAC were divided into groups of 5–6 animals. NGF at a dose of 104 μg/kg, СVF at a dose of 250 μg/kg and ketoprofen at a dose of 40 mg/kg were administered intraperitoneally each 3rd–4th day over 12 days; two groups of animals received NGF and ketoprofen or NGF and СVF at doses indicated above. Drugs were dissolved in saline. The control group received saline. Each animal received three treatments. The volume of developed subcutaneous tumor was assessed as in [3]. The tumor tissue was taken from animals at day 12 after inoculation; cryostat sections for histochemical studies were prepared, stained by hematoxylin–eosin and analyzed as in [39], and the blood samples were collected for preparation of serum. Sera of intact mice were also obtained.

### 5.4. Immunoenzyme Analysis (ELISA) 

Immunoenzyme analysis (ELISA) was performed with DuoSet ELISA Mouse IL-1β/IL-1F2, DuoSet ELISA TGF-β1 and DuoSet ELISA TNF-α assay kits (R & D Systems, Minneapolis, MN, USA). Serum levels of IL-1β, TGF-β1 and TNF-α were determined according to the manufacturer’s protocol. Optical density values at 450 nm were measured using a BioTek ELx808 microplate reader (BioTek, Winooski, VT, USA). The concentration of samples was calculated according to the corresponding OD value and the concentration of the standard substance.

### 5.5. Enzymatic Activity Determination

The activities of LDH and SDH were determined by the tetrazolium method according to [40], using the computer data-processing program Image J (National Institutes of Health, Bethesda, MD, USA). Each measurement was performed on more than 300 individual tumor cells obtained from three to five animals from each group.

### 5.6. Statistical Analysis

Statistical analysis of the tumor volume, cytokine serum levels and enzymatic activity was performed with the Mann–Whitney test. The differences were considered significant for *p* values <0.05. All results are presented as the mean ± SEM (standard error of the mean).

All studies involving animals were approved by the Commission on Bioethics of the Institute of Physiology of the National Academy of Sciences of Belarus, established in accordance with Order No. 44 of 07.06.2013, consisting of: Chairman—Head of the Laboratory A. Yu. Molchanova; Deputy Chairman—Deputy Director for Scientific and Innovation S. V. Mankovskaya; Members—head of the laboratory of physiotherapy and balneology, E.I. Kalinovskaya; chief scientific worker, L.I. Archakova; research worker, A.E. Pyzh; senior research worker, V.S. Lukashevich; research worker, S.B. Kohan; scientific secretary of the Institute of Physiology—N.F. Pavlova; secretary—T.E. Kuznetsova. The ethical approval code is No. 2. The date of approval is 7 August, 2014.
